# QSAR Study of p56^lck^ Protein Tyrosine Kinase Inhibitory Activity of Flavonoid Derivatives Using MLR and GA-PLS

**DOI:** 10.3390/ijms9091876

**Published:** 2008-09-22

**Authors:** Afshin Fassihi, Razieh Sabet

**Affiliations:** Department of Medicinal Chemistry, Faculty of Pharmacy, Isfahan University of Medical Sciences and Health Services, 81746-73461, Isfahan, Iran. E-Mail: Sabet@pharm.mui.ac.ir

**Keywords:** Protein tyrosine kinase, Flavonoid, QSAR, Chemometrics, SED analysis

## Abstract

Quantitative relationships between molecular structure and p56^lck^ protein tyrosine kinase inhibitory activity of 50 flavonoid derivatives are discovered by MLR and GA-PLS methods. Different QSAR models revealed that substituent electronic descriptors (SED) parameters have significant impact on protein tyrosine kinase inhibitory activity of the compounds. Between the two statistical methods employed, GA-PLS gave superior results. The resultant GA-PLS model had a high statistical quality (R^2^ = 0.74 and Q^2^ = 0.61) for predicting the activity of the inhibitors. The models proposed in the present work are more useful in describing QSAR of flavonoid derivatives as p56^lck^ protein tyrosine kinase inhibitors than those provided previously.

## 1. Introduction

The quantitative structure-activity relationship (QSAR) research field provides medicinal chemists with the ability to predict drug activity by mathematical equations which construct a relationship between the chemical structure and the biological activity [1, 2]. These mathematical equations are in the form of y = Xb+e that describe a set of predictor variables (X) with a predicted variable (y) by means of a regression vector (b) [3]. After the earlier QSAR studies by Hansch, who showed a correlation between biological activity and octanol-water partition coefficient [2], it is now assumed that the sum of substituent effects on the steric, electronic and hydrophobic interaction of compounds with their receptor determines their biological activity [4–6]. The first step in constructing the QSAR models is finding one or more molecular descriptors that represent variation in the structural property of the molecules by a number [7]. Nowadays, a wide range of descriptors are being used in QSAR studies which can be classified into different categories according to the Karelson approach including; constitutional, geometrical, topological, quantum, chemical and so on [8]. There are different variable selection methods available including; multiple linear regression (MLR), genetic algorithm (GA), principal component or factor analysis (PCA/FA) and so on. The mathematical relationships between molecular descriptors and activity are used to find the parameters affecting the biological activity and/or estimate the property of other molecules.

It is now well established that protein tyrosine kinases (PTKs) provide a central switching mechanism in cellular signal transduction pathways by catalyzing the transfer of the γ-phosphate of either ATP or GTP to specific tyrosine residues in certain protein substrates [9, 10]. This regulatory control plays a crucial role in signal transduction pathways that regulate several cellular functions under both normal and deregulated conditions [11–14]. PTKs are the intracellular effectors for many growth hormone receptors. After the discovery of activated PTKs as the product of dominant viral-transforming genes (oncogenes) providing the early hypothesis for the connection between protein tyrosine phosphorylation and cell transformation, enough evidence are now available to suggest that inappropriate or elevated expression of PTKs contribute to the transformed state of cells in many human malignancies [15–19]. P56^lck^ is a lymphoid-specific protein tyrosine kinase that is principally expressed in T lymphocytes [20]. Association of p56lck with the cytoplasmic tail of various cell surface receptors, as well as associations of p56lck with intracellular targets of phosphorylation, suggests that this tyrosine kinase plays a central role in coordinating early signal transduction events [21]. Based on this knowledge it is clear that, substances which can modulate the activity of PTKs might be potentially effective therapeutic agents. The key step in the mechanism of kinase activity of all PTKs is the recognition and binding of a nucleoside triphosphate (usually ATP) and an appropriate tyrosyl-containing substrate to the enzyme. Direct transfer of phosphate between the two molecules is the next step in the PTKs function [22]. A variety of compounds can inhibit the function of PTKs in a manner which is competitive with respect to nucleotide binding. Among such competitive inhibitors are flavonoids, a group of low molecular weight plant natural products that include one of the largest classes of naturally-occurring polyphenolic compounds [23, 24]. This group of plant natural products is largely responsible for the colors of many fruits and flowers, and over 4,000 flavonoid pigments have been characterized and classified according to their chemical structure. Chemically they are C6-C3-C6 compounds in which the two C6 groups are substituted benzene rings, and the C3 group is an aliphatic chain which contains a pyran ring. Flavonoids occur as O-or C-glycosides or in the “free” state as aglycones with hydroxyl or methoxyl groups present on the aglycone. The flavonoids may be divided into seven types: flavones, flavonols, flavonones, chalcones, xanthones, isoflavones, and biflavones. Flavonoids have been gained wide interest as potential pharmacological agents since some of the best sources of flavonoids are foods: apples, blueberries, bilberries, onions, soy products and tea. Furthermore numerous medicinal plants contain therapeutic amounts of flavonoids, which are used to treat a wide variety of disorders [25].

Here, we consider the inhibitory activity of flavonoids against protein–tyrosine kinase p56^lck^. Several QSAR studies were reported on this class of molecules using different descriptors and different methods of modeling. Thakur *et al.* described a QSAR study on p56^lck^ protein tyrosine kinase inhibitor flavonoids using only hydration energy and hydrophobic parameters [26]. Nikolovska-Coleska *et al.* treated a set of 104 derivatives with standard linear regression technique by the use of classical/quantum descriptors [27]. The same dataset was treated by Novic *et al.* with a counter propagation neural network by the use of classical/quantum descriptors [28]. Oblak *et al.* applied a wide variety of descriptors with CODESSA software on the above-mentioned dataset [29]. A quantum chemical/classical QSAR study on a set of 75 flavonoids and closely related compounds tested as p56^lck^ protein tyrosine kinase and AR inhibitors has been carried out by Stefanic *et al.* and the obtained structure-activity relationships of both enzyme systems were compared [30]. A comprehensive *ab initio* study of 3D structures of some flavonoids is reported by Meyer [31]. Deeb *et al.* calculated nodal orientation with program NODANGLE [32].

In the present paper, the QSAR study for a series of 50 flavonoid analogues with the ability to inhibit protein tyrosine kinase has been considered [32]. In a comprehensive study of the PTK system we used a very large descriptor set (more than 600 topological, geometrical, constitutional, functional group, electrostatic, quantum and chemical descriptors) and different analyses: Hansch, Free-Wilson and substituent electronic descriptors (SED), in order to be able to compare the predictive ability of descriptors from different descriptor groups. Multiple linear regression (MLR) and genetic algorithm partial least squares (GA-PLS) methods were applied as methods for modeling.

## 2. Results and Discussion

The structural features and biological activity of the studied compounds are listed in Table 1. Calculated descriptors for each molecule are summarized in Table 2.

### 2.1. MLR analysis

In the first step, separate stepwise selection-based MLR analyses were performed using different types of descriptors, and then, an MLR equation was obtained utilizing the pool of all calculated descriptors. The results are summarized in Table 3. Correlation coefficient (r^2^) matrix for the descriptors used in different MLR equations is shown in Table 4. Collinear descriptors degrade the performance of MLR equations and such models have lowered prediction ability.

In Table 3 the QSAR models derived for different derivatives by using different sets of molecular descriptors are listed. Table 3 provides the resulted equations for the studied compounds. The first equation of Table 3 was found by using chemical descriptors (E_1_). This equation explained the negative effect of hydration energy and molecular weight (Mass) of molecules on protein tyrosine kinase inhibitory activity. Equation E_2_ shows that among quantum descriptors, most positive charge (MPC) has a negative effect on protein tyrosine kinase inhibitory activity and reveals the presence of columbic interactions between the ligands and receptors. The negative sign of the coefficient of MPC demonstrates that ligands with the least MPC could interact with receptor more efficiently. This indicates that there is probably a negative region in receptor which produces columbic interactions with ligand. Equation E_3_ of Table 3 demonstrates the effect of constitutional descriptors. It includes the negative effects of average molecular weight (AMW), number of multiple bonds (nBM) and number of aromatic bonds (nAB) on protein tyrosine kinase inhibitory activity. Molecules with lower coefficient of AMW show better protein tyrosine kinase inhibitory activity and decreasing the number of multiple bonds of compounds results in activity enhancement. The MLR equation of Table 3 was obtained from the pool of topological descriptors (E_4_) explained the positive effect of mean information content on the distance equality (ICR), path/walk 4-randic shape index (PW4), average connectivity index chi-4 (X4v) and the negative effect of mean information content vertex degree magnitude (IVDM) and average valence connectivity index chi-1 (X1v) on protein tyrosine kinase inhibitory activity. This equation describes the structure-activity relationship better than those obtained from the chemical, quantum, constitutional descriptors.

The equation obtained from the effect of geometrical parameter on protein tyrosine kinase inhibitory activity of the studied compounds has been described as E_5_ of Table 3. It explains the positive effect of spherosity (SPH) and negative effect of sum of geometrical distances between N...O, *i.e.* G (N...O) on protein tyrosine kinase inhibitory activity. The effect of functional groups on protein tyrosine kinase inhibitory activity of the studied compounds has been described by equation E_6_ of Table 3. This three-parametric equation does not have a high statistical quality, which suggests that the protein tyrosine kinase inhibitory activity of the studied molecules is not highly dependent on the type of functional group; but it is dependent on the structural changes induced by variations in functional groups. The negative sign of nNO_2_ and nOHt indicates that molecules with lower number of nitro groups (aliphatic) and tertiary alcohols (aliphatic) bind to protein kinase stronger. On the other hand, number of hydroxyl groups (nOH) represents direct effect on the inhibitory activity of the compounds. The Hansch equation (E_7_) shows the importance of steric, electronic and lipophilic factors on protein tyrosine kinase inhibitory activity. These factors are described by L_3_ (Length parameter of C_3_ substituent), ℑR′_3_, ℑR_8_ (Swain and Lupton field parameter of C-R′_3_ and C-R_8_ substitutes) and π_5_ (lipophilic parameter of C_5_ substitute), respectively. The negative coefficient of π_5_ indicates that lipophilic substituents at R_5_ are not favorable for binding affinity. This equation shows the positive effect of ℑR′_3_ and the negative effect of ℑR_8_ on the inhibitory activity of the compounds. In addition the negative effect of L_3_ describes that the presence of bulky groups at C_3_ leads to decreased activity because bulky groups hinder strong interaction between ligands and the enzyme. The SED equation (E_8_) shows the importance of SED factors on protein tyrosine kinase inhibitory activity. One of the parameters is molecular orbital energy HOMO_A_3 (Highest occupied molecular orbital parameter of C_3_ substitute) and the other one is SNQ8 (Sum of negative charges parameter of C_8_ substitute). It explains the positive effect of HOMO_A_3 and negative effect of SNQ8 on protein tyrosine kinase inhibitory activity.

The last Equation (E_9_) was obtained from the all types of calculated descriptors. Stepwise selection and elimination of variables produced a four-parametric QSAR equation. This equation shows that geometrical (SPH), quantum (MPC), Hansch (L_3_) and SED (SNQ8) parameters are major factors that affect protein tyrosine kinase inhibitory activity of compounds. Among these descriptors MPC and L_3_ have negative effects and the others have positive effects on the protein tyrosine kinase inhibitory activity.

### 2.2. Free-Wilson analysis

The simple Free-Wilson analysis (FWA) was considered to indicate which substituents on ring **B** and chromone moiety contribute to protein tyrosine kinase inhibitory activity and which ones detract from activity [33]. As indicated in Table 1, the molecules used in this study have a phenyl ring (ring **B**) and chromone moiety with different types of substituents in different positions of the ring. Some important substituents such as methoxyl, hydroxyl and amine are used in calculations. Therefore, the descriptors data matrix built for the FWA has 44 rows (i.e., number of selected molecules for FWA) and 24 columns (i.e., three substituents at eight substitution positions on the flavonoid structure). The elements of the descriptor data matrix are 1 or 0, to indicate the presence or absence of a given substituent in a specified position in a molecule, respectively. The following two-parametric equation was found between the activity data (y) and the Free-Wilson type descriptors data matrix:

1 pIC50=3.893 (±0.089)+0.439(±0.207)R'3_Hydroxyl − 1.103 (±0.534)R5_Methoxyl                       R2=0.70,  N=44,  F=31.45,  SE=0.30

Equation (1) describes that protein tyrosine kinase inhibitory activity of studied compounds is directly affected by the presence of electron-donating hydroxyl group in the *meta* position (R′_3_) of the phenyl ring and most probably this part of the flavonoid molecule interacts with the catalytic domain of the enzyme. The same result was obtained by other researchers [27]. A methoxyl group on C-R_5_ detracts from the inhibitory activity, according to this equation.

### 2.3. GA-PLS analysis

**Figure 1.**
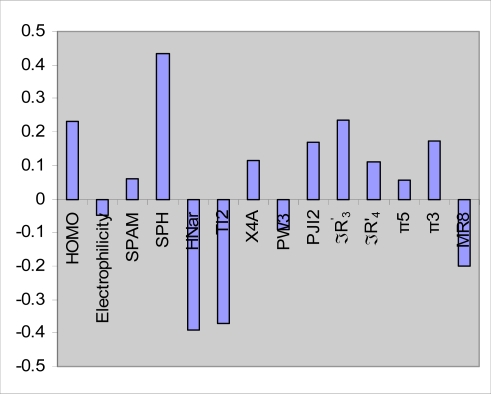
PLS regression coefficients for the variables used in GA-PLS model.

In PLS analysis, the descriptors data matrix is decomposed to orthogonal matrices with an inner relationship between the dependent and independent variables. Therefore, unlike MLR analysis, the multicolinearity problem in the descriptors is omitted by PLS analysis. Because a minimal number of latent variables are used for modeling in PLS; this modeling method coincides with noisy data better than MLR. In order to find the more convenient set of descriptors in PLS modeling, genetic algorithm was used. To do so, many different GA-PLS runs were conducted using different initial set of populations. The data set (n = 50) was divided into two group: calibration set (n = 40) and prediction set (n = 10). Given 40 calibration samples; the leave-one out cross-validation procedure was used to find the optimum number of latent variables for each PLS model. The most convenient GA-PLS model that resulted in the best fitness contained 14 indices, four of them being those obtained by MLR. The PLS estimate of coefficients for these descriptors are given in Figure 1. As it observed, a combination of quantum, topological, geometrical and Hansch descriptors have been selected by GA-PLS to account the protein tyrosine kinase inhibitory activity of flavonoid derivatives. The majority of these descriptors are topological indices. The resulted GA-PLS model possessed a high statistical quality R^2^ = 0.74 and Q^2^ = 0.61. The predictive ability of the model was measured by applying to 10 external test set molecules. The squared correlation coefficient for prediction was 0.82 and standard error of prediction was 0.30. The values of pIC_50_ using GA-PLS model (refined from cross-validation or external prediction set) along with the corresponding relative errors of prediction (REP) are shown in Table 1. Very small values of relative errors (between ± 0.40) confirm the accuracy of the proposed GA-PLS model for modeling protein tyrosine kinase inhibitory activity of the studied flavonoid derivatives.

**Figure 2.**
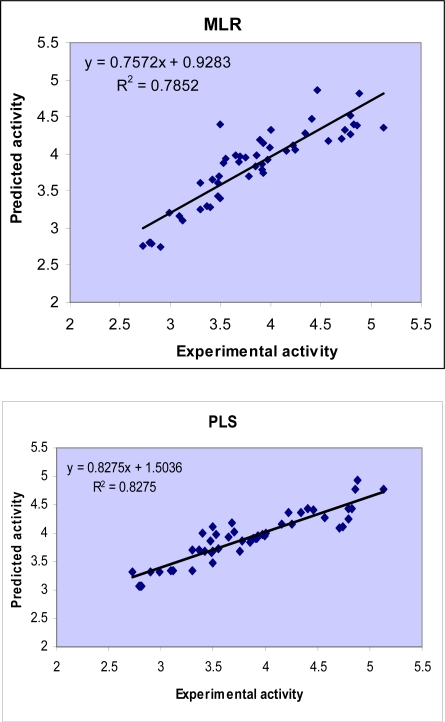
Plots of the cross-validated predicted activity against the experimental activity for the QSAR models obtained by MLR, GA-PLS methods.

Comparison between the results obtained by GA-PLS and MLR methods indicates higher accuracy of GA-PLS method in describing the inhibitory activity of flavonoid derivatives toward protein tyrosine kinase enzyme. The difference in accuracy of the two regression methods used in this study is visualized in Figure 2 by plotting the predicted activity (by cross-validation) against the experimental values. Obviously, two linear models represented scattering of data around a straight line with slope close to one. As it is observed, the plot of data resulted by GA-PLS represents the lowest scattering and the plot obtained by MLR analysis (which is obtained from E_9_) is in the second order of accuracy.

**Figure 3.**
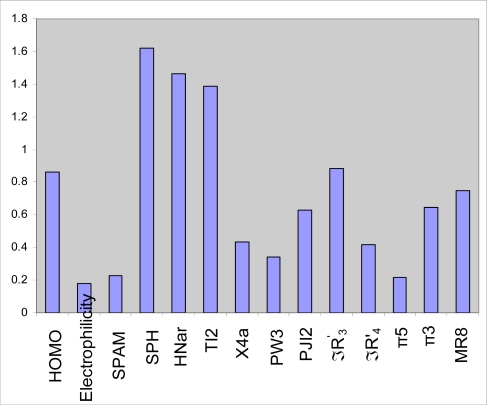
Plot of variables important in projection (VIP) for the descriptors used in GA-PLS model.

To measure the significance of the 14 selected PLS descriptors in the protein tyrosine kinase inhibitory activity; VIP was calculated for each descriptor [34]. The VIP analysis of PLS equation is shown in Figure 3. VIP shows that HNar and TI2, which are topological, and SPH which is a geometrical parameter, are the most important indices in the QSAR equation derived by PLS analysis. In addition, quantum parameters such as (HOMO) and Hansch (ℑR′_3_) have been found to be moderately influential parameters.

## 3. Methodology

### 3.1. Software

The two-dimensional structures of molecules were drawn using Hyperchem 7.0 software. The final geometries were obtained with the semi-empirical AM1 method in Hyperchem program. The molecular structures were optimized using the Polak-Ribiere algorithm until the root mean square gradient was 0.01 kcal mol^−1^. The resulted geometry was transferred into Dragon program package, which was developed by Milano Chemometrics and QSAR Group [35]. The z-matrix of the structures was provided by the software and transferred to the Gaussian 98 program. Complete geometry optimization was performed taking the most extended conformation as starting geometries. Semi-empirical molecular orbital calculation (AM1) of the structures was preformed using Gaussian 98 program [36].

### 3.2. Activity data & descriptor generation

The biological data used in this study are protein tyrosine kinase inhibitory activity, −log (IC_50_), of a set of 50 flavonoid analogues [32]. The structural features and biological activity of these compounds are listed in Table 1 and then used for subsequent QSAR analysis as dependent variables. The large number of molecular descriptors was calculated using Hyperchem, Dragon package and Gaussian 98. Some chemical parameters including molecular volume (V), molecular surface area (SA), hydrophobicity (Log P), hydration energy (HE) and molecular polarizability (MP) were calculated using Hyperchem Software. The Dragon software calculated different functional groups, topological, geometrical and constitutional descriptors for each molecule. Gaussian 98 was employed for calculation of different quantum chemical descriptors including, dipole moment (DM), local charges, and HOMO and LOMO energies. Hardness (η), softness (S), electronegativity (χ) and electrophilicity (ω) were calculated according to the method proposed by Thanikaivelan *et al*. [37]. Classical substituent constants including hydrophobic constant (π), the Hammet electronic constants (σ), the Taft field effect (FI), resonance (R) substituent and steric (molar refractivity MR and STERIMOL) constants were also used as descriptor in this study [38]. The calculated descriptors for each molecule are summarized in Table 2.

### 3.3. Data screening & model building

The selected descriptors from each class and the experimental data were analyzed by the stepwise regression SPSS (version 12.0) software. The calculated descriptors were collected in a data matrix whose number of rows and columns were the number of molecules and descriptors, respectively. Multiple linear regression (MLR) and partial least squares (PLS) were used to derive the QSAR equations and feature selection was performed by the use of genetic algorithm (GA). The resulted models were validated by leave-one out cross-validation procedure (using MATLAB software) to check their predictability and robustness. However, this procedure did not produce good results and therefore we used genetic algorithm (GA-PLS) to select the best variables.

Application of PLS allows the construction of larger QSAR equations, while still avoiding over-fitting and eliminating most variables. PLS is normally used in combination with cross-validation to obtain the optimum number of components [39, 40]. The PLS regression method used in this study was the NIPALS-based algorithm existed in the chemometrics toolbox of MATLAB software (version 7.1 Math work Inc.). Leave-one-out cross-validation procedure was used to obtain the optimum number of factors based on the Haaland and Thomas F-ratio criterion [41].

### 3.4. Variable importance in the projection (VIP)

In order to investigate the relative importance of the variable appeared in the final model obtained by GA-PLS method, variable important in projection (VIP) was employed [34]. VIP values reflect the importance of terms in PLS model. According to Erikson *et al.* X-variables (predictor variables) could be classified according to their relevance in explaining y (predicted variable), so that VIP > 1.0 and VIP < 0.8 mean highly or less influential, respectively, and 0.8 < VIP< 1.0 means moderately influential [8].

### 3.5. Substituent electronic descriptors (SED)

Electronic descriptors obtained from quantum chemical calculations have found major popularity and there is a challenge between calculation complexity and accuracy to select the quantum chemical calculation methods (i.e., semi-empirical and *ab initio*) [42]. To simplify the quantum chemical calculations Hemmateenejad *et al.* recently have hypothesized that the calculations could be performed on the substituents instead of whole molecular structures and the resulting electronic features can be considered as electronic descriptors which have found major popularity in QSAR/QSPR studies [43,44]. Hemmateenejad *et al.* proposed substituent electronic descriptors (SED) as an alternative to both substituent constants and molecular descriptors [43]. SED analysis for each substituent was used in our study and the calculated descriptors are listed in Table 2. They can be classified into three different electronic categories including local charges, dipoles and orbital energies. Since most of the constituents are open shell quantum species (due to being in doublet quantum state as a radical molecule), a difference in energy between two electronic energy populations, alpha (spine up) and beta (spine down) can be seen using Gaussian 98. It provides some additional descriptors HOMO_A_, HOMO_B_, LUMO_A_, LUMO_B_, HAD, HD_B_, SOF_A_, SOF_B_, EN_A_, EN_B_, EPH_A_, and EPH_B_ stem from two different alpha and beta electronic population energy, where the subscript A and B stand for alpha and beta population of electronic energy, respectively. Therefore, a total of 26 electronic descriptors were calculated for each substituent.

## 4. Conclusions

Quantitative relationships between molecular structure and protein tyrosine kinase inhibitory activity of flavonoid derivatives were discovered by two chemometrics methods: MLR and GA-PLS. Different QSAR models revealed that SED parameters have significant impact on protein tyrosine kinase inhibitory activity of the compounds. In this series a significant role of topological and geometrical parameters on the inhibitory activity was observed. Using the pool of all types of calculated descriptors a new QSAR model was derived for these compounds. In this model the importance of quantum, geometrical, SED and Hansch parameters have an effect on protein tyrosine kinase inhibitory activity was indicated. A comparison between the two statistical methods employed indicated that GA-PLS represented superior results. The resulted GA-PLS model possessed a high statistical quality (R^2^ = 0.74 and Q^2^ = 0.61) for predicting the activity of the inhibitors. The models proposed in present work are more useful in describing QSAR of flavonoid derivatives as p56^lck^ protein tyrosin kinase Inhibitors than those proposed previously.

## Figures and Tables

**Table 1.** Chemical structure of flavonoid derivatives used in this study and their experimental and predicted activity for protein kinase inhibition. 
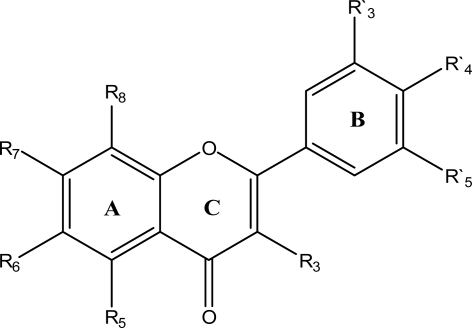 Chemical structure of flavonoid derivatives.

Compound	R	Experimental pIC_50_^a^	Predicted pIC_50_	REP b
**1**	5,7-OH,4′-NH_2_	5.13	4.7707	−0.0753
**2**	3,5,7,3′,4′-OH	4.88	4.9431	0.0128
**3**	3,7,3′,4′-OH	4.86	4.7707	−0.0187
**4**	5,7,4′-OH	4.83	4.4356	−0.0889
**5**	5,4′-OH	4.80	4.2603	−0.1267
**6**	6,3′-OH	4.80	4.4242	−0.0849
**7**	6-OH,5,7,4′-NH_2_	4.74	4.1061	−0.1544
**8**	5,7-OH	4.71	4.0895	−0.1518
**9**	4′-OH,3′,5′-OCH_3_	4.57	4.2687	−0.0706
**10**	5,7,3′,4′-OH	4.46	4.4172	−0.0097
**11**	7,3′-OH	4.41	4.4358	0.0058
**12**	6-OH,5,7,3′-NH_2_	4.34	4.3681	0.0064
**13**	6-OMe,8,3′-NH_2_	4.25	4.1649	−0.0204
**14**	6-OH,3′,4′,5′-OCH_3_	4.22	4.3591	0.0319
**15**	3,5,7,4′-OH,3′,5′-OCH_3_	4.16	4.1649	0.0012
**16**	3,5,7,3′,5′-OH	4.00	3.9947	−0.0013
**17**	6,4′-NH_2_	3.99	3.9613	−0.0072
**18**	6,8,4′-NH_2_	3.97	3.9764	0.0016
**19**	6-OH,8,4′-NH_2_	3.93	3.9446	0.0037
**20**	6,4′-OH	3.93	3.9247	−0.0013
**21**	7,8,4′-OH,3′,5′-OCH_3_	3.92	3.8990	−0.0054
**22**	8,4′-NH_2_	3.91	3.8994	−0.0027
**23**	6,4′-OH,3′,5′-OCH_3_	3.89	3.9133	0.0060
**24**	7-OH,4′-NH_2_	3.86	3.8815	0.0056
**25**	7-OH,6,4′-NH_2_	3.85	3.8296	−0.0053
**26**	7,4′-OH	3.78	3.8621	0.0213
**27**	7,8,3′OH	3.75	3.6903	−0.0162
**28**	6,3′-NH_2_	3.70	4.0228	0.0803
**29**	4′-NH_2_	3.68	4.1850	0.1207
**30**	5-OH,6,4′-NH_2_	3.65	3.9325	0.0718
**31**	3,5,7-OH	3.53	3.9794	0.1129
**32**	5,4′-OH,7-OCH_3_	3.55	3.7315	0.0487
**33**	5,3′-OH	3.50	4.1209	0.1507
**34**	7,8-OH	3.50	3.4873	−0.0036
**35**	5-OH,8,4′-NH_2_	3.49	3.6705	0.0492
**36**	7-OH,8,4′-NH_2_	3.48	3.6694	0.0516
**37**	7-OH	3.47	3.8567	0.1003
**38**	6-OCH_3_,8,4′-NH_2_	3.43	3.6709	0.0683
**39**	7,8-OH,3′,4′,5′-OCH_3_	3.40	4.0058	0.1512
**40**	3-COOCH_3_,4′-OH	3.36	3.7081	0.0939
**41**	4′-OH	3.30	3.7081	0.1101
**42**	7-OH,6,3′-NH_2_	3.30	3.3419	0.0125
**43**	7-OH,6,8,4′-NH_2_	3.12	3.3419	0.0664
**44**	3-COOCH_3_,4′-NH_2_	3.09	3.3419	0.0754
**45**	3-COOH,7-OCH_3_,4′-OH	2.99	3.3262	0.1011
**46**	7,4′-OH,3′,5′-OCH_3_	2.90	3.3262	0.1281
**47**	7-OH,6,8,4′-NO_2_	2.81	3.0674	0.0839
**48**	3-COOH,4′-OH	2.80	3.0674	0.0872
**49**	5-OCH_3_,8,4′-NH_2_	2.79	3.0674	0.0904
**50**	7-OH,8,4′-NO_2_	2.73	3.3262	0.1793

a pIC_50_ = –log (IC_50_),

b REP = Relative Error Prediction

**Table 2.** Brief description of some descriptors used in this study.

Descriptor type	Molecular Description
Constitutional	Molecular weight, no. of atoms, no. of non-H atoms, no. of bonds, no. of heteroatoms, no. of multiple bonds (nBM), no. of aromatic bonds, no. of functional groups (hydroxyl, amine, aldehyde, carbonyl, nitro, nitroso, etc.), no. of rings, no. of circuits, no of H-bond donors, no of H-bond acceptors, no. of Nitrogen atoms (nN), chemical composition, sum of Kier-Hall electrotopological states (Ss), mean atomic polarizability (Mp), number of rotable bonds (RBN), mean atomic Sanderson electronegativity (Me), etc.
Topological	Molecular size index, molecular connectivity indices (X1A, X4A, X2v, X1Av, X2Av, X3Av, X4Av), information content index (IC), Kier Shape indices, total walk count, path/walk-Randic shape indices (PW3, PW4, Zagreb indices, Schultz indices, Balaban J index (such as MSD) Wiener indices, topological charge indices, Sum of topological distances between F..F (T(F..F)), Ratio of multiple path count to path counts (PCR), Mean information content vertex degree magnitude (IVDM), Eigenvalue sum of Z weighted distance matrix (SEigZ), reciprocal hyper-detour index (Rww), Eigenvalue coefficient sum from adjacency matrix (VEA1), radial centric information index, 2D petijean shape index (PJI2), etc.
Geometrical	3D petijean shape index (PJI3), Gravitational index, Balaban index, Wiener index, etc.
Quantum	Highest occupied Molecular Orbital Energy (HOMO) , Lowest Unoccupied Molecular Orbital Energy (LUMO), Most positive charge (MPC), Least negative charge (LNC), Sum of squares of charges (SSC), Sum of square of positive charges (SSPC), Sum of square of negative charges (SSNC), Sum of positive charges (SUMPC), Sum of negative charges (SUMNC), Sum of absolute of charges (SAC), Total dipole moment (DM_t_), Molecular dipole moment at X-direction (DM_X_), Molecular dipole moment at Y-direction (DM_Y_), Molecular dipole moment at Z-direction (DM_Z_), Electronegativity (χ= −0.5 (HOMO-LUMO)), Electrophilicity (ω= χ^2^/2 η) ,Hardness (η = 0.5 (HOMO+LUMO)), Softness (S=1/η).
Functional group	Number of total tertiary carbons (nCt), Number of H-bond acceptor atoms (nHAcc), number of total hydroxyl groups (nOH), number of unsubstituted aromatic C(nCaH), number of ethers (aromatic) (nRORPh), etc.
Chemical	LogP (Octanol-water partition coefficient), Hydration Energy (HE), Polarizability (Pol), Molar refractivity (MR), Molecular volume (V), Molecular surface area (SA).
Substituent electronic descriptors	RMSQ (Root mean square error of charges), SPQ ( Sum of positive charges), SNQ ( Sum of negative charges), RMSDM (Root mean square of dipole moments at any Cartesian coordinate direction), TDM (Total dipole moment), FRMS (Root mean square force that any atom in constituent molecule see right before the optimization), FMAX (Maximum force on molecule), HOMO (Highest occupied molecular orbital), LUMO (Lowest unoccupied molecular orbital), HD (Hardness), SOF (Softness), EPH (Electrophilicity), EN (Electronegativity).

**Table 3.** The results of MLR analysis with different types of descriptors.

No.	Descriptor source	MLR Equations	N	R^2^	SE	RMS_CV_	Q^2^	F
E_1_	Chemical	pIC_50_ = 4.893 (± 0.735) − 0.056 (± 0.017) HE −0.007 (± 0.003) Mass	50	0.40	0.55	0.58	0.32	13.82
E_2_	Quantum	pIC_50_ = 6.362 (± 0.565) − 6.805 (± 1.505) MPC	50	0.43	0.53	0.54	0.38	17.44
E_3_	Constitutional	pIC_50_ = 3.139 (± 1.250) − 0.438 (± 0.100) nBM − 0.506 (± 0.205) AMW − 0.584 (± 0.266) nAB	50	0.49	0.49	0.51	0.42	19.65
E_4_	Topological	pIC_50_ = 17.242 (± 0.605) − 3.374 (± 0.545) IVDM − 53.95 (± 12.355) X1Av + 2.349 (± 0.696) ICR +24.874 (±9.569) PW4 + 73.575 (±33.719) X4A	50	0.72	0.38	0.48	0.58	30.13
E_5_	Geometrical	pIC_50_ = −15.093 (± 3.339) + 19.450 (± 3.406) SPH − 0.010 (± 0.002) G(N...O)	50	0.60	0.43	0.47	0.49	17.23
E_6_	Functional group	pIC_50_ = 3.672 (± 0.123) − 0.414 (± 0.130) nNO_2_ −1.098 (± 0.369) nOH_t_ + 0.160 (± 0.058) nOH	50	0.53	0.45	0.50	0.45	12.67
E_7_	Hansch	pIC_50_ = 4.219 (± 0.289) − 0.615 (± 0.202) π_5_ + 1.462 (± 0.555) ℑR′_3_ − 1.379 (± 0.490) ℑR_8_ −0.249 (± 0.111) L_3_	50	0.53	0.45	0.50	0.45	12.67
E_8_	SED	pIC_50_ = −0.708 (± 1.228) − 9.570 (± 2.500) HOMO_A_3 + 1.092 (±0.308) SNQ8	50	0.82	0.32	0.30	0.61	51.43
E_9_	Molecular descriptor	pIC_50_ = −19.763 (± 4.304) − 4.785 (± 1.275) MPC + 25.113 (± 4.142) SPH + 0.849 (± 0.264) SNQ8 − 0.357 (± 0.136) L_3_	50	0.83	0.31	0.28	0.62	52.43

**Table 4.** Correlation coefficient (r^2^) matrix for the descriptors of flavone derivatives used in the MLR equation.

	HE	Mass	MPC	nBM	AMW	nAB	ASP	G(N...O)	X1A_V_	ICR	PW4	X4A	IVDM	nNO2	nOHt	nOH	ℑR′_3_	L_3_	ℑR_8_	π_5_	pIC_50_
**HE**	1	−0.234	0.192	0.124	−0.327	0.236	−0.006	0.00	0.651	0.075	−0.012	0.316	0.065	0.069	0.047	−0.745	−0.394	0.067	−0.005	0.485	−0.347
**Mass**		1	0.531	0.580	0.512	0.136	−0.269	0.328	−0.655	0.416	0.541	−0.631	0.816	0.554	0.099	0.211	0.326	0.196	0.487	0.040	−0.268
**MPC**			1	0.953	0.715	0.366	−0.233	0.623	−0.539	0.304	0.050	−0.329	0.904	0.876	0.259	−0.227	−0.286	0.289	0.595	0.156	−0.547
**nBM**				1	0.778	0.165	−0.094	0.725	−0.624	0.390	0.016	−0.325	0.937	0.972	0.114	−0.196	−0.211	0.125	0.687	0.193	−0.498
**AMW**					1	0.050	−0.200	0.356	0.897	0.037	0.116	−0.206	0.718	0.775	0.116	0.434	0.136	0.125	0.620	0.065	−0.191
**nAB**						1	−0.684	−0.127	0.069	−0.192	0.257	−0.397	0.235	−0.073	0.692	−0.086	−0.198	0.930	−0.108	0.185	−0.364
**ASP**							1	0.294	0.155	0.538	0.532	0.388	−0.221	0.069	0.369	−0.273	−0.201	−0.768	−0.039	−0.098	0.269
**G(N...O)**								1	−0.379	0.578	0.299	0.348	0.618	0.763	−0.138	−0.478	−0.437	−0.182	0.508	0.034	−0.329
**X1AV**									1	−0.130	−0.171	0.413	−0.651	−0.647	−0.052	−0.572	−0.270	−0.056	−0.542	0.229	0.058
**ICR**										1	−0.212	−0.277	0.442	0.441	−0.104	−0.410	−0.161	−0.278	0.153	0.168	−0.080
**PW4**											1	−0.157	0.261	−0.045	0.158	0.336	0.413	0.356	0.029	−0.249	0.002
**X4A**												1	−0.489	−0.233	−0.252	−0.046	−0.025	−0.466	−0.261	−0.157	0.347
**IVDM**													1	0.891	0.155	−0.100	−0.030	0.218	0.663	0.192	−0.494
**nNO2**														1	−0.050	−0.177	−0.166	−0.097	0.720	0.151	−0.416
**nOHt**															1	0.061	−0.137	0.513	−0.075	0.128	−0.306
**nOH**																1	0.621	0.104	−0.004	−0.375	0.370
ℑ**R′3**																	1	−0.070	0.008	−0.014	0.315
**L3**																		1	−0.143	0.085	−0.259
ℑ**R8**																			1	0.224	−0.367
**π5**																				1	−0.451
**pIC50**																					1
